# Changes in Microbiome Dominance Are Associated With Declining Lung Function and Fluctuating Inflammation in People With Cystic Fibrosis

**DOI:** 10.3389/fmicb.2022.885822

**Published:** 2022-05-13

**Authors:** Dario L. Frey, Calum Bridson, Susanne Dittrich, Simon Y. Graeber, Mirjam Stahl, Sabine Wege, Felix Herth, Olaf Sommerburg, Carsten Schultz, Alexander Dalpke, Marcus A. Mall, Sébastien Boutin

**Affiliations:** ^1^Translational Lung Research Center (TLRC), Member of the German Center for Lung Research (DZL), Heidelberg, Germany; ^2^Department of Translational Pulmonology, University of Heidelberg, Heidelberg, Germany; ^3^Department of Infectious Diseases, Medical Microbiology and Hygiene, University of Heidelberg, Heidelberg, Germany; ^4^Department of Pneumology and Critical Care Medicine, Thoraxklinik at the University Hospital Heidelberg, Heidelberg, Germany; ^5^Division of Pediatric Pulmonology and Allergology and Cystic Fibrosis Center, Department of Pediatrics, University of Heidelberg, Heidelberg, Germany; ^6^Department of Pediatric Respiratory Medicine, Immunology and Critical Care Medicine and Cystic Fibrosis Center, Charité-Universitätsmedizin Berlin, Berlin, Germany; ^7^Berlin Institute of Health (BIH), Berlin, Germany; ^8^German Center for Lung Research (DZL), Associated Partner Site, Berlin, Germany; ^9^Department of Chemical Physiology and Biochemistry, Oregon Health & Science University, Portland, OR, United States; ^10^Institute of Medical Microbiology and Virology, Technische Universität Dresden, Dresden, Germany

**Keywords:** microbiome, cystic fibrosis, 16S rRNA gene, inflammation, longitudinal study

## Abstract

Airway inflammation and microbiome dysbiosis are hallmarks of cystic fibrosis (*CF*) lung disease. However, longitudinal studies are needed to decipher which factors contribute to the long-term evolution of these key features of *CF.* We therefore evaluated the relationship between fluctuation in microbiome and inflammatory parameters in a longitudinal study including a short- (1-year) and a long-term (3+ years) period. We collected 118 sputum samples from 26 *CF* adult patients and analyzed them by 16S rRNA gene sequencing. We measured the levels of inflammatory cytokines, neutrophil elastase, and anti-proteinases; lung function (FEV1% predicted); and BMI. The longitudinal evolution was analyzed based on (i) the rates of changes; (ii) the intra-patient stability of the variables; and (iii) the dependency of the rates of changes on the baseline values. We observed that the diversity of the microbiome was highly variable over a 1-year period, while the inflammatory markers showed a slower evolution, with significant changes only observed in the 3+ year cohort. Further, the degree of fluctuation of the biomass and the dominance of the microbiome were associated with changes in inflammatory markers, especially IL-1β and IL-8. This longitudinal study demonstrates for the first time that the long-term establishment and periodical variation of the abundance of a dominant pathogen is associated with a more severe increase in inflammation. This result indicates that a single time point or 1-year study might fail to reveal the correlation between microbial evolution and clinical degradation in cystic fibrosis.

## Introduction

Cystic fibrosis (*CF*) is caused by a mutated cystic fibrosis transmembrane conductance regulator (*CFTR*) gene, leading to a deficient chloride, bicarbonate and fluid transport across the apical surface of the epithelial organs (Mall and Hartl, 2014). Consequently, people with *CF* (PwCF) have hyper-concentrated mucus in their lungs, which fosters the establishment of chronic inflammation and infection (Elborn, 2016). Therefore, the microbiome of PwCF is of major interest in the field. The development of techniques, such as next-generation sequencing, has made microbiome studies affordable and manageable in many hospitals, and they are now becoming, along with extended cultures, a part of the precision medicine tools to assess patients’ disease status (Boutin and Dalpke, 2017; Héry-Arnaud et al., 2019).

The lung microbiome is established early after birth, and it has been demonstrated that a microbial signature can already be found *in utero* (Al Alam et al., 2020). In PwCF, studies have shown that the lung microbiome is polymicrobial in nature rather than determined by a monospecific infection, as previously thought (Tunney et al., 2008; Lopes et al., 2015). Later in life, during disease progression, a microbiome dominated by a single taxon may evolve as a sign of chronic infection (Boutin et al., 2017). In addition to airway dysbiosis, chronic inflammation is present from early life (Balázs and Mall, 2019). PwCF suffer from chronic neutrophilic inflammation, which is exaggerated by the infection and is a key factor in the progression of *CF* lung disease (Tirouvanziam, 2006; Giacalone et al., 2020).

A lot of our understanding of the microbiome structure in *CF* has come from cross-sectional studies. However, large differences in microbiome composition, inflammation, and infection between patients can mask patterns. These limitations can be overcome by using longitudinal studies, which allow for comparisons of changes in microbiome and inflammation relative to the patient baseline, and also control for patient-specific confounding factors. Most of the previous longitudinal studies have focused on the detailed examination of pulmonary exacerbations, the effects of antibiotic treatment, or early-life development (Cox et al., 2010; Zhao et al., 2012; Whelan et al., 2017; Ahmed et al., 2019; Cuthbertson et al., 2020; Hahn et al., 2020; Raghuvanshi et al., 2020). In general, these studies have found declining bacterial α-diversity with declining lung function and increased antibiotic use (Zhao et al., 2012; Cuthbertson et al., 2020; Raghuvanshi et al., 2020). However, there is still a lack of understanding of the relationship between the lung microbiome and inflammation over time. In a recent cross-sectional study including >100 patients (Frey et al., 2021a), we showed that dominance of the lung microbiota by a single species was associated with the higher levels of airway inflammation. In order to build on the previous study, and further investigate the relationship between the microbiota, inflammation, and lung function, we designed an observational longitudinal study comparing the changes in the microbiota and inflammation over 3+ years to their variation over 1-year in a cohort of 26 PwCF. In contrast to our previous cross-sectional study, the design allows us to investigate changes in individual patients over time and has the advantage that each patient has their own baseline, ensuring the accurate measure of the extent and progression of changes in microbiota, inflammation and lung function.

## Materials and Methods

### Study Cohort

**Figure 1:**
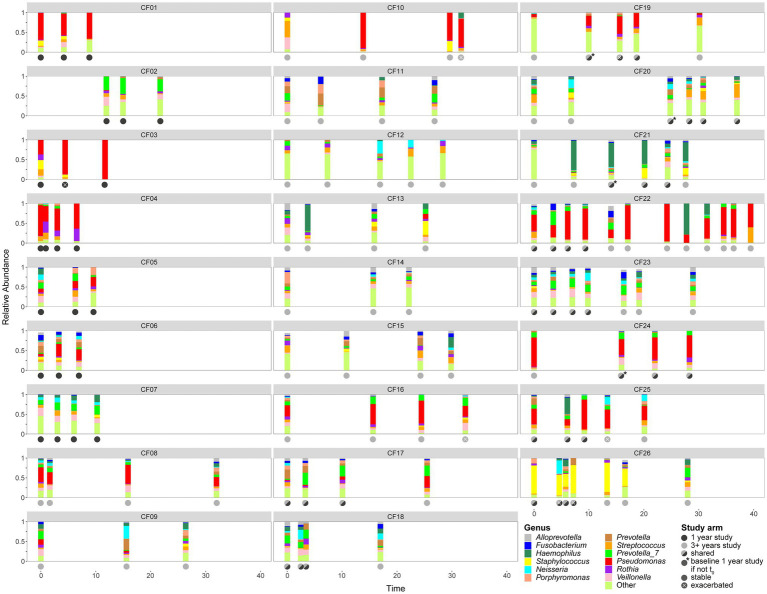
Relative abundance for the 12 most common genera depicted as a stacked bar plot for each visit grouped by patient. The x-axis indicates time in months since the baseline, the y-axis shows relative abundance. Dots underneath the stacked bar plot are color-coded according to the study arm they were assigned to, dark gray: 1-year, light gray: 3+ years. Baseline visits is defined as the first routine visit without exacerbation or aggravating symptoms if different from *t*_0_ the visits are indicated with a white dot, stable visits are solid dots and exacerbated visits are indicated with a white X. If several factors are true, they are plotted over each other.

This study was approved by the Ethics Committee of the University of Heidelberg, and the written informed consent was obtained from all the patients (S-370/2011). Details on the diagnosis of *CF*, sputum sampling, and pulmonary function testing are provided in the Supplementary Material. The cohort consisted of 26 PwCF and 118 samples, with the samples collected over a 4-year period. The cohort was split into two subgroups to understand how fast the correlation between microbiota changes and inflammation was established, one consisting of ≥3 visits per patient within 1-year, referred to as the 1-year cohort (*n* = 17), and the other of patients having ≥1 visit per year over ≥3 years, termed as the 3+ year cohort (*n* = 19), with 10 patients included in both study arms (Figure 1). Patients were treated according to the standard of care (Castellani et al., 2018). Details on demographics, CFTR genotype, pancreatic insufficiency, and antibiotic use are provided in Table 1 and in Supplementary Table S1 and Supplementary Figure S1.

**Table 1:** Clinical characteristics of the 1-year and 3+ year cohort.

		1-Year	3+ Years
Number of subjects	*n*	17	19
Number of visits	*n (mean)*	57^#^ (3.35)	95^#^ (5.00)
Age (years)^*^	Median (range)	29.03 (17.73–61.43)	26.84 (17.73–71.51)
Sex	Females/males (*n*)	4/13	5/14
BMI (kg/m^2^)^*^	Median (range)	21.73 (17.30–30.32)	20.16 (17.27–28.02)
FEV_1_% predicted^*^	Median (range)	52.94 (17.49–89.67)	48.81 (17.41–108.9)
CFTR genotype			
F508del/F508del	*n* (percentage)	9 (53%)	7 (37%)
F508del/other	*n* (percentage)	5 (29%)	9 (47%)
Other/other	*n* (percentage)	3 (18%)	3 (16%)
CFTR modulator			
Lumacaftor/Ivacaftor	*Patients* (visits)	2 (5)	2 (16)
Tezacaftor/Ivacaftor	*Patients* (visits)	3 (9)	3 (6)
Pancreatic insufficiency	*n* (percentage)	15 (88%)	17 (89%)
Antibiotic treatment			
Inhaled	*Patients* (visits)	6 (26)	6 (47)
Oral	*Visits*	---	2
IV	*Visits*	2	2
Mixed treatment	*Patients* (visits)	9 (17)	12 (33)
Inhaled/oral	*Visits*	13	23
Inhaled/IV	*Visits*	1	4
Inhaled/oral/IV	*Visits*	3	6
None	*Patients* (visits)	2 (11)	1 (9)
Unknown	*Visits*	1	2

* At baseline visit.

# Thirty-four visits are shared.

BMI, body mass index; FEV_1_% predicted, forced expiratory volume in 1 s percent predicted; CFTR, cystic fibrosis transmembrane conductance regulator; and IV, intravenous.

### Sputum Collection and Sample Pre-treatment for Inflammatory Biomarker Analysis

In total, 118 sputum samples were included in the study and treated as previously described (Frey et al., 2021a). In brief, the spontaneously expectorated sputum was aliquoted into two parts: one part was used for the microbiota study, and the other part was used for the measurement of inflammatory biomarkers. For the cytokine and NE activity measurements, sputum samples were treated as previously described (Dittrich et al., 2018; Frey et al., 2021a). Details are provided in the Supplementary Material.

### Inflammatory Biomarker Analysis

A panel of previously described (Bonfield et al., 1995) pro-inflammatory cytokines (IL-1β, IL-6, IL-8, and TNF-α), which are released by inflammatory cells as a response to various microorganisms and foster inflammatory process propagating the lung tissue destruction, were quantified using a commercially available cytometric bead array (BD Biosciences, San Diego, CA, United States) according to the manufacturer’s instructions. Levels of endogenous anti-proteases (SLPI, A1AT/NE complex, and TIMP1) were measured by commercially available enzyme-linked immunosorbent assays (R&D Systems, Minneapolis, MN, United States and eBioscience, Frankfurt am Main, Germany) according to the manufacturers’ instructions. Free NE activity was measured using the FRET reporter NEmo-1 (Gehrig et al., 2014; Sirius Fine Chemicals, Bremen, Germany) in cell-free supernatants as previously described (Dittrich et al., 2018; Frey et al., 2021a,b). The number of neutrophils in sputum was determined by differential cell counts of May-Grünwald-Giemsa–stained cell preparations. Details are provided in the Supplementary Material.

### Sample Pre-treatment and Microbiome Analysis

For the microbiota study, samples were processed as previously described (Boutin et al., 2015). In short, samples were treated with PMA™ dye (Biotium Inc., Hayward, United States), DNA was extracted and library was prepared using two PCR reaction to amplify the V4 region of the 16S rRNA gene and ligate to Illumina primers with barcodes to be sequenced on a Miseq instrument (2*300 cycles). In parallel, the copy number of 16S rRNA gene was quantified by qPCR to evaluate the biomass. Details are provided in the Supplementary Material.

The sequence data were processed using Dada2 to produce amplicon sequence variants (ASVs; Callahan et al., 2016). For each sample, the bacterial community was characterized by calculating α-diversity (Hill numbers when *q* = 0 and *q* = 1), dominance (relative abundance of the most abundant ASV) and β-diversity (weighted UniFrac distance using relative abundance data). PERMANOVA was used to investigate whether the lung bacterial community differed among patients. Mixed-effects models were constructed between time, taxon abundance and each of the microbiota diversity, clinical and inflammation parameters. The models were either linear mixed-effects models (LMMs), generalized linear mixed-effects models (GLMMs with β-distribution) or zero-inflated Gaussian mixed-effects models (ZIGMM) depending on which best fit the data. The relationship between the microbiota diversity, clinical and inflammation parameters was determined by calculating Spearman’s rank correlations between: (a) their rates of change over time within a patient (regression coefficients of their relationship with time); (b) their intra-patient variability (mean pairwise distance between all samples within a patient); and (c) correlations of the value of α-diversity and the FEV_1_% predicted at the patient baseline with the rate of change of the variables. For all analyses, the Benjamini-Hochberg method was used to control for multiple comparisons. Further details are in the Supplementary Material.

## Results

### The Microbiome Structure of Each Patient Is Personalized and Variable Over Time

We observed a high level of individualization in the microbiome composition of each patient (Figure 1). Interestingly in contrast to the previous studies on other patients from the same *CF* study cohort (Boutin et al., 2017; Frey et al., 2021a), the number of patients characterized by *Staphylococcus* sp. or atypical pathogens such as *Achromobacter* or *Stenotrophomonas* is limited. Most of the patients are characterized by either a *P. aeruginosa* dominated microbiome or a quite diverse microbiome with mostly commensals.

### The Microbiome Diversity Decreases Over the 1-Year Period While Lung Function and Inflammation Are Not Significantly Altered

**Figure 2:**
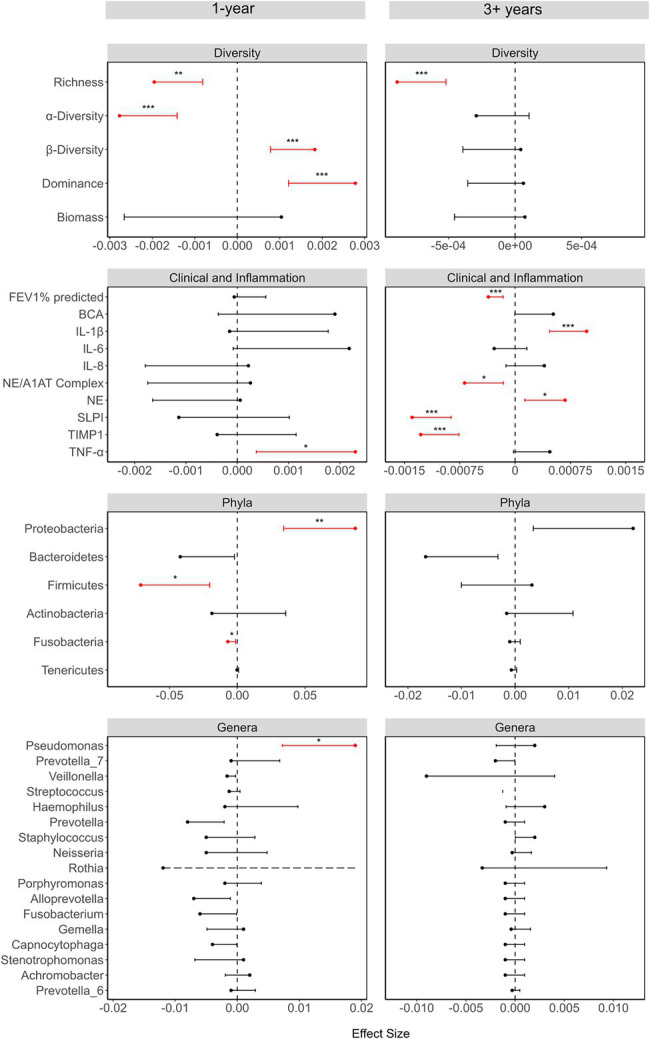
Summary of the linear models investigating the relationship between each variable and time, showing the effect sizes, 95% CI and values of *p* (as asterisks). The effect sizes and values of *p* were calculated using either linear mixed-effects models (LMMs), generalized linear mixed models (GLMMs), or zero-inflated Gaussian models (ZIGMMs) as outlined in the Supplementary Methods. Where variables were transformed before use in a model, the effects sizes are from the models with the transformed data. For the LMMs, the response variables were standardized, by subtracting the mean and dividing by the standard deviation, for ease of comparison. For the taxonomic variables, time was converted to months rather than days. Only the error bars going toward zero are showing to maintain a scale that aids visualization. The dashed error bar for Rothia in the 1-year study represents the fact that the confidence interval extends beyond the limits of the figure, but the figure was cut to maintain a good scale for comparison. The values of *p* of phyla and genera were adjusted for multiple comparisons using the Benjamini-Hochberg method. Values of *p* indicated as: ^*^<0.05, ^**^≤0.01, and ^***^≤0.001.

**Figure 3:**
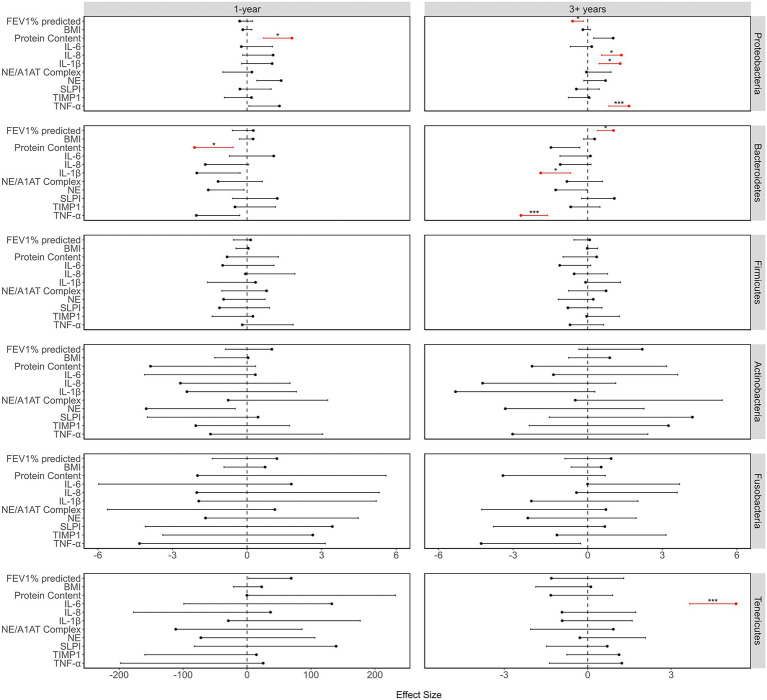
A summary of the relationship between the relative abundances of the most abundant phyla and the clinical and inflammation parameters, showing the effect sizes, 95% CI and values of *p*. Effect sizes and values of *p* were determined using LMMs, and the values of *p* were adjusted within each set of tests of a response variable, using the Benjamini-Hochberg method. Prior to running the models, the response variables were standardized, by subtracting the mean and dividing by the SD, for ease of comparison. Only the error bars going toward zero are showing to maintain a scale that aids visualization. Values of *p* indicated as: ^*^<0.05, and ^***^≤0.001.

There was a strong correlation of the microbiome diversity parameters with time over a 1-year period, but very little correlation of inflammation or clinical parameters with time. There was a decrease in richness (LMM: value of *p* = 0.002) and α-diversity (LMM: value of *p*<0.001), while both β-diversity (GLMM: value of *p* = 0.002) and dominance (GLMM: value of *p*<0.001) increased over time (Figure 2). Furthermore, there were increases in Proteobacteria and declines in Bacteroidetes, Firmicutes, and Fusobacteria, while at the genus level, *Pseudomonas* (ZIGMM: Adjusted value of *p* = 0.049) increased with time. Of the clinical and inflammatory parameters, only TNF-α (LMM: value of *p* = 0.026) showed a relationship with time. The only correlation of phyla with inflammation and clinical parameters over a 1-year period, was the significant correlation of Proteobacteria and Bacteroidetes abundance with protein content (LMM: respectively: adjusted value of *p* = 0.026; 0.042; Figure 3).

### The Microbiome-Inflammation Relationship Is Not Strong Over the 1-Year Period

**Figure 4:**
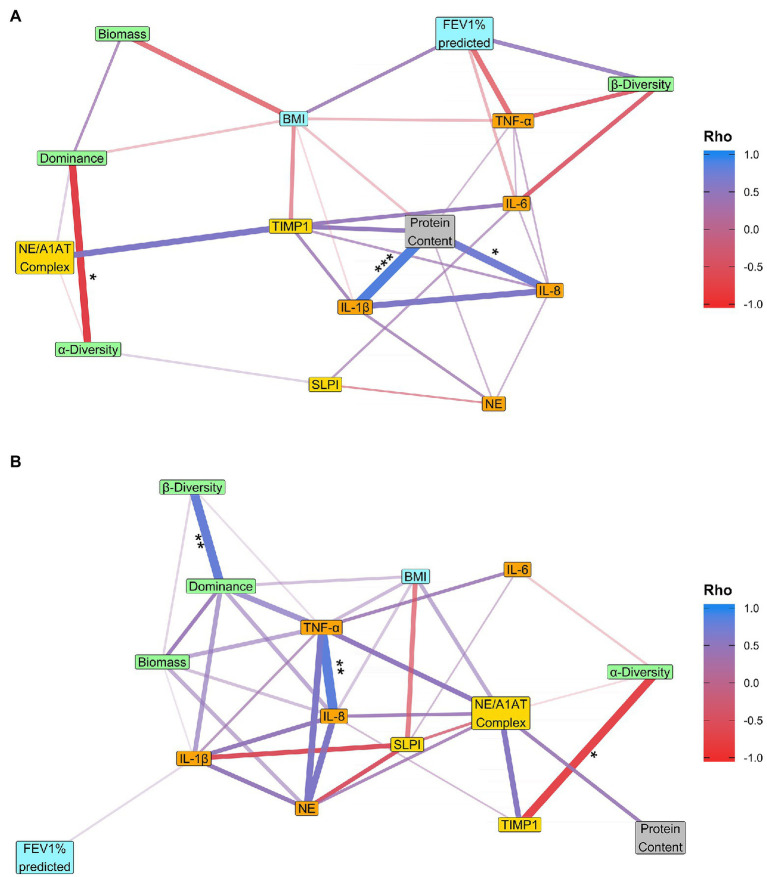
Correlations between variables in the 1-year cohort. **(A)** In terms of rate of change of a variable over time. For each variable, the relationship between that variable and time was determined within each patient, and the resulting regression coefficients were correlated across variables. **(B)** The intra-patient stability. For each variable, intra-patient stability was calculated as the average pairwise distance between samples within a patient. These average distances were then correlated across variables. Microbiota are given in green, clinical in blue, pro-inflammatory in orange, anti-protease in yellow, and general parameters in gray. The size of the Spearman’s rank correlation coefficient (Rho) is represented by the width and color of the edges, and only correlations with a Rho greater than 0.3 or smaller than −0.3 are shown. Values of *p* were adjusted using the Benjamini-Hochberg method. Values of *p* indicated as: ^*^<0.05, ^**^≤0.01, and ^***^≤0.001.

There was no correlation between microbiota diversity parameters and clinical or inflammation parameters over a 1-year period (Figure 4A) for any measure of change, except for a negative correlation between α-diversity stability and TIMP1 stability (Spearman’s rank: Adjusted value of *p* = 0.032; Figure 4B). However, there were significant correlations between microbiota diversity parameters. There was a negative correlation between dominance and α-diversity in terms of rates of change (Spearman’s rank: adjusted value of *p* = 0.015). This means that patients who showed an increase in dominance over a year, also showed a decrease in α-diversity, and vice versa. There was also a positive correlation between the intra-patient stabilities of β-diversity and dominance (Spearman’s rank: adjusted value of *p* = 0.001). Furthermore, the baseline α-diversity was negatively correlated with the rate of change in α-diversity over time (Spearman’s rank: adjusted value of *p* = 0.003) and the intra-patient stability of α-diversity (Spearman’s rank: adjusted value of *p* = 0.003; Supplementary Figure S2).

### The Long-Term Period Highlights Temporal Inflammatory Changes While the Microbiome Stabilizes

In contrast to the 1-year cohort, over 3+ years there were strong correlations of inflammation and clinical parameters with time, but there were no correlations between microbiome diversity parameters and time, with the exception of species richness (LMM: value of *p*<0.001; Figure 2). IL-1β (LMM: value of *p* < 0.001), NE (LMM: value of *p* = 0.018), NE/A1AT complex (LMM: value of *p* = 0.013), SLPI (LMM: value of *p* < 0.001), and TIMP1 (LMM: value of *p* < 0.001) were correlated with time, and FEV_1_% predicted (LMM: value of *p* < 0.001) was found to decline with time in the 3+ year period. TNF-α showed a weak trend toward an increase with time. Furthermore, compared to the 1-year cohort, there was a greater association of phyla abundance with clinical and inflammation parameters. Proteobacteria showed a positive correlation with both TNF-α (LMM: Adjusted value of *p* < 0.001) and IL-1β (LMM: Adjusted value of *p* = 0.012) and a negative correlation with FEV_1_% predicted (LMM: Adjusted value of *p* = 0.025), while Bacteroidetes showed the opposite trend (Figure 3). Additionally, there was a positive correlation between Proteobacteria relative abundance and IL-8 (LMM: adjusted value of *p* = 0.012), and Tenericutes abundance was positively associated with IL-6 (LMM: adjusted value of *p* < 0.001).

### The Fluctuation of the Microbiome Structure and Diversity Is Associated With Inflammation and Lung Function Over the Long-Term Period (3+ Year)

**Figure 5:**
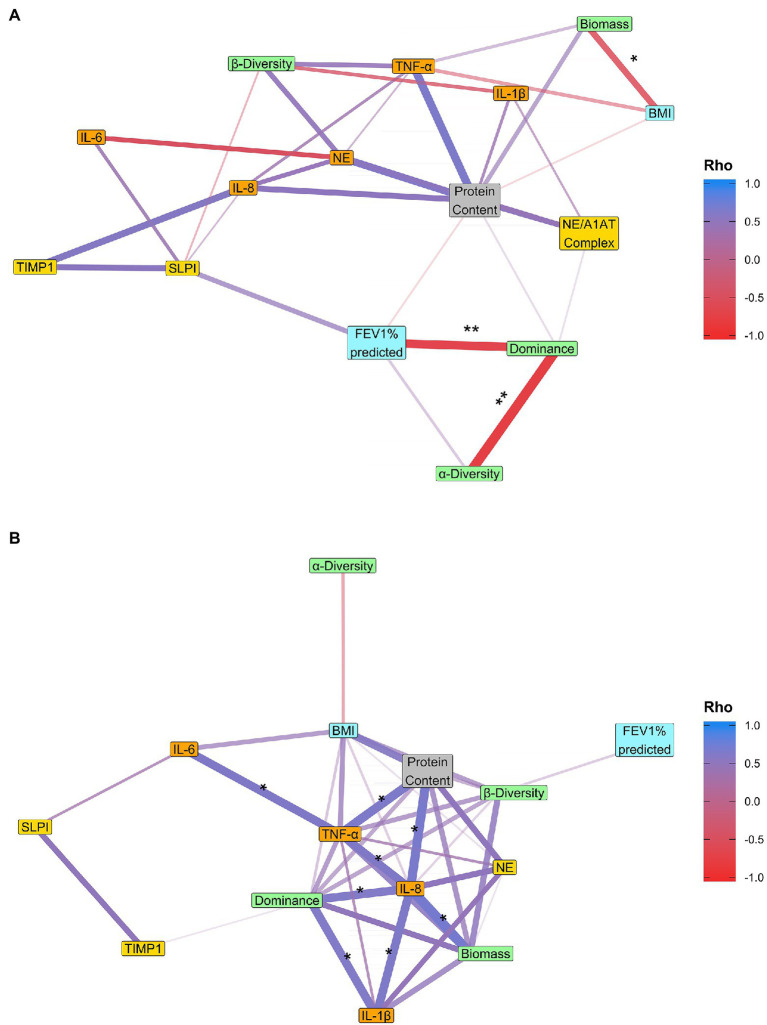
Correlations between variables in the 3+ year cohort. **(A)** In terms of rate of change of a variable over time. For each variable, the relationship between that variable and time was determined within each patient, and the resulting regression coefficients were correlated across variables. **(B)** The intra-patient stability. For each variable, intra-patient stability was calculated as the average pairwise distance between samples within a patient. These average distances were then correlated across variables. Microbiota are given in green, clinical in blue, pro-inflammatory in orange, anti-protease in yellow, and general parameters in gray. The size of the Spearman’s rank correlation coefficient (Rho) is represented by the width and color of the edges, and only correlations with a Rho greater than 0.3 or smaller than −0.3 are shown. Values of *p* were adjusted using the Benjamini-Hochberg method. Values of *p* indicated as: ^*^<0.05 and ^**^≤0.01.

Larger increases in dominance over 3+ years were correlated with larger declines in FEV_1_% predicted (Spearman’s rank: adjusted value of *p* = 0.008; Figure 5A). In contrast, the relationship between the microbiome and inflammation parameters was more dynamic whereby fluctuations in the microbiota were associated with fluctuations in levels of inflammation. In particular, patients with highly fluctuating levels of dominance, also had highly fluctuating levels of both IL-1β and IL-8 (Spearman’s rank: both adjusted value of *p* = 0.043; Figure 5B). The variability in dominance within a patient was not significantly related to the number of hospital visits in which they received antibiotics (linear model: inhaled antibiotics: *p* = 0.367, oral: *p* = 0.339, IV: *p* = 0.196). Furthermore, like with dominance, variability in biomass within a patient was positively correlated with variability in IL-8 (Spearman’s rank: adjusted value of *p* = 0.043), meanwhile the rate of change in biomass was negatively correlated with rate of change in BMI (Spearman’s rank: adjusted value of *p* = 0.046). In terms of correlations between microbiota diversity parameters, there was a negative correlation between the rate of change in dominance and the rate of change in α-diversity (Spearman’s rank: Adjusted value of *p* = 0.003), but unlike the 1-year cohort there were no significant correlations between dominance and β-diversity for any measure of change. There were also fewer significant correlations with the baseline condition in the 3+ year cohort compared to the 1-year cohort. The only correlation was between baseline α-diversity and intra-patient α-stability (Spearman’s rank: adjusted value of *p* = 0.037; Supplementary Figure S2). As dominance was mostly related to *Pseudomonas aeruginosa* abundance, we performed the analysis to only include patients with no samples dominated by *Pseudomonas*. We observed still a strong negative correlation between the rates of change of dominance and FEV_1_% predicted (Rho = −0.673, *p* = 0.078), although it was marginally non-significant due to the smaller sample size. There was also an increase in the number of negative correlations between dominance and inflammation, suggesting dominance generally can have an influence on inflammation. However, in the absence of *Pseudomonas aeruginosa*, we see weaker correlations of dominance stability with IL8 stability (Rho = 0.3, *p* = 0.814) and IL1-β stability (Rho = 0.5, *p* = 0.498) and with much higher values of *p*. Thus, the relationship between dominance and inflammation stability is potentially driven by variability in *Pseudomonas* dominance (Supplementary Figures S3 and S4).

### Effect of Exacerbation and Antibiotic Use

Although not the focus of the study, we investigated whether exacerbated visits or visits where patients were receiving antibiotics were related to the microbiome or clinical parameters, using the mixed effects models outlined above. However, due to the lack of exacerbation events, we cannot draw strong conclusions about their role. In the 3+ year period, *Neisseria* abundance, *Staphylococcus* abundance and richness are related to exacerbation, total antibiotic use and oral antibiotic use, respectively. The results are summarized in Supplementary Table S2.

## Discussion

Of the microbiome diversity parameters tested, only dominance was found to correlate with lung function and inflammation, which may suggest changes in overall microbiota composition are not as important as changes in abundance of the dominant taxa. We found that steeper increases in dominance were associated with steeper declines in lung function; for the first time, we showed that periodic variation in dominance was correlated with changes in inflammation. Furthermore, the microbiome strongly correlated with time in the 1-year cohort, which indicates dynamic changes in the microbiome over 1-year, as has been observed in the previous studies (Raghuvanshi et al., 2020). The microbiome was more stable over 3+ years, but there were stronger correlations with lung function and inflammation than over 1-year.

The difference between the correlation of the microbiome parameters with time in the 1 and 3+ years studies can be explained by the mechanisms governing the acquisition of the microbiome in the lower airways. The colonization of the lower airways by the upper airway microbiome will trigger a cycle of colonization/elimination, causing variability over time within a short period (Dickson et al., 2015). There is also the potential for exacerbation and antibiotic use to influence the microbiome, but we do not find evidence for this in the 1-year cohort. However, there were a low number of exacerbations in both cohorts (1-year: *n* = 4, 3+ years: *n* = 6).

The microbiome was more stable over the 3+ year period, except for a decline in species richness. However, increases in inflammation (IL-1β and NE), as well as decreases in anti-proteases (SLPI and TIMP1) and lung function, were only observable within the 3+ year cohort, indicating that those processes are more gradual and establish themselves slowly. There were also stronger correlations of the microbiome with lung function and inflammation in the 3+ year cohort compared to the 1-year cohort. Our data showed that increased dominance was associated with decreased lung function in the 3+ year cohort, which supports results from previous cross-sectional studies (Cuthbertson et al., 2020; Frey et al., 2021a). There is an established link between α-diversity and lung function (Whelan et al., 2017; Raghuvanshi et al., 2020; Frey et al., 2021a), but here we show for the first time in a longitudinal study, that the relationship is driven by changes in the dominant taxa rather than changes in species richness.

A second important finding is that intra-patient variation in dominance is strongly correlated with variability in inflammation (Figure 5). This indicates, along with the lack of correlation between α-diversity and inflammation, that the increase in relative abundance of one specific species at a certain timepoint is more crucial than the decrease in α-diversity in regard to the corresponding increase in inflammation (Figure 5). This finding suggests that the increase in the immune response (especially through IL-8 and IL-1β) will be more visible during the phase of a new infection or re-establishment of an infection when the abundance of the pathogen will rise within the microbiota. This could be explained by a change in the virulence of the pathogen itself or a change in the transcriptome of the microbiome during re-infection. These results will have to be validated with transcriptional approaches, but the effects of increased dominance highlight the importance of infection screening/management to try to stop the increase in pathogen dominance or virulence and thus mitigate the impact on inflammation and lung function decline. Furthermore, a recent study of highly persistent variants of *P. aeruginosa* demonstrated that patients who harbor highly persistent variants are less likely to have successful eradication, which leads to a dominance of the persistent variant (Bartell et al., 2020). The variability in dominance did not seem to be related to antibiotic use, as intra-patient stability in dominance was not related to a patient’s antibiotic regime. The fact that the study was longitudinal enabled us to show that it is the variability in dominance and inflammation that is correlated, and that there is no linear relationship over time. This result would have been missed by a cross-sectional study.

The increase in dominance was explained by the increase in Proteobacteria (especially *Pseudomonas*) and the decrease in Bacteroidetes and Firmicutes. The temporal changes in the phyla Proteobacteria and Bacteroidetes were correlated with lung function and the changes in inflammation. PwCF that showed an increase in Proteobacteria were prone to exhibit more inflammation and a steeper decline in lung function over 3+ years. This is in line with findings published in the cross-sectional studies (Zemanick et al., 2015; Hahn et al., 2020), in which patients with a more diverse microbiome had better lung function (Boutin et al., 2017; Frey et al., 2021a). The concordance between our study and cross-sectional studies is due to a change in the microbiome from a commensal-rich community with Bacteroides and Firmicutes as prevalent members to a Proteobacteria-dominated microbiome which is one sign of infection with typical *CF* pathogens (Cox et al., 2010; Zhao et al., 2012; Zemanick et al., 2015, 2017; Boutin et al., 2017, 2018; Jorth et al., 2019; Cuthbertson et al., 2020). We highlight, in a longitudinal setting, the negative impact of infection by Proteobacteria on the establishment of chronic inflammation as well as on lung function in PwCF. The re-analysis excluding the patient with *Pseudomonas* dominance showed that the correlation is not exclusively driven by *Pseudomonas* but that *Pseudomonas* is a major driver of the relationship between dominance and inflammation. The impact of *Pseudomonas* on our results can be explain by the fact that it is the main *CF* pathogen in young adult with *CF* and it is the most prevalent one in our cohort. Our re-analysis show that other pathogen might have the same impact without reaching significance due to the sample size. Therefore, it would be important to validate our results with a cohort of PwCF dominated with other pathogens such as *Staphylococcus aureus*, *Burkholderia cepacia complex*, *Stenotrophomonas*.

Our study also had limitations because of its longitudinal design. First, a longitudinal analysis of only 1-year is too short to find a significant association between microbiome data and clinical outcome. This is due to the variability across the microbiome and the lack of significant changes in inflammation and lung function over such a short period, despite the high number of samples per year (≥ 3), which is a lot given the fact that the study was based on routine check-ups which are only scheduled 3–4 times a year. Furthermore, the design aimed for this study lead us to exclude many patients from our cohort that were not producing sputum three times per year or at least once every year for 3+ years. Therefore, we have a small cohort size compared to a cross-sectional study. We also showed that the initial starting point of the longitudinal study influenced the results. Patients with lower initial α-diversity or lower initial lung function will not show temporal evolution in those parameters, as they already represent an “end-stage” status. However, the association appears to be non-linear, as there is a smaller effect on the microbiota when lung function decreases from a high to middle than middle to low, as described before (Zhao et al., 2012). Since patients with a mid-level FEV_1_% predicted value already show a highly dysbiotic microbiome with a dominant infection, the microbiome cannot be altered much more, yet lung function can still be affected. Furthermore, the low number of exacerbations in our dataset does not allow us to make conclusions about the impact of exacerbation despite the statistical significance, but we see an influence of antibiotic treatment on both inflammation and, to a lesser extent, the microbiome (especially *Neisseria* abundance). Our study is also limited regarding taxonomic changes because we used a 16S rRNA-based metagenomic approach due to cost-effectiveness of the technique in regards to the high number of samples (*n* = 118), which meant taxa could only be differentiated to the genus level. Further our methodology could not capture the viral and fungal taxa present, unlike Mac Aogáin et al. (2021). However, the fungal and viral components of the microbiome are without a doubt an important factor influencing inflammation. Further, the extraction of functional data is also not possible; to discriminate on a functional basis, whole-genome sequencing would be necessary (Sherrard et al., 2016; Lamoureux et al., 2019). Moreover, sputum can have high geographical variability that might also have influenced the temporal variability observed in our study, in contrast to broncho-alveolar lavage or bronchial brushes, although it is less invasive and can therefore be performed more often (Raghuvanshi et al., 2020). Thus, we cannot ensure that the 1-year variability is not a reflection of spatial heterogeneity in the lung. In comparison, FEV_1_% predicted is a global measure of lung function and is used as the gold standard in the *CF* field. Both parameters showed significant correlations over the examined study period in the 3+ year study cohort. We also have to acknowledge that the two cohorts do not overlap perfectly and only 10 patients are present in both the 1 year and 3+ years cohorts. This fact limits the possibility to really compare the short-term and long-term changes but does not affect the correlation observed for each cohort. Finally, we used spontaneous sputum, so we had a slightly biased cohort limited to patients who could produce spontaneous sputum in a constant enough manner to be included in the longitudinal setting.

In summary, our study showed that despite the variability in the microbiome over a 1-year period, which is due to the ecology of the lung microbiome and/or clinical events such as exacerbation and antibiotic usage, the relationship between the microbiome and inflammation is established over a period of 3+ years. Our data identify that the long-term establishment of dominance by Proteobacteria is associated with a more severe increase in inflammation (especially IL-8 and IL-1β) and, more importantly, that it is the instability of the dominance that correlates with inflammation and lung function.

## Data Availability Statement

The data presented in the study are deposited in the NCBI repository under the bioproject number PRJNA785129 and the scripts used for analysis are available under the link https://figshare.com/s/de66d2124dc8687c1d97.

## Ethics Statement

The studies involving human participants were reviewed and approved by the Ethics Committee of the University of Heidelberg. Written informed consent to participate in this study was provided by the participants’ legal guardian/next of kin.

## Author Contributions

DF, SB, SD, and MAM designed the study. DF and SB collected the samples and processed them. SB and CB performed the bioinformatic analysis. DF, SB, and CB prepared the results, visualization, and the first draft. DF, SD, SG, MS, SW, OS, FH, CS, AD, MAM, and SB critically reviewed and revised the manuscript. All authors contributed to the article and approved the submitted version.

## Funding

This study was supported by grants from the German Ministry for Education and Research (82DZL00401, 82DZL004A1, and 82DZL009B1 to MAM, AD, and CS, 82DZL004B1 to SB), the European Commission (Seventh Framework Program Project No. 603038, CFMatters to MAM), the German Cystic Fibrosis Association Mukoviszidose e. V. (project number 1605 to SD, project number 1805 to AD and SB), the Deutsche Forschungsgemeinschaft (DFG, German Research Foundation—SFB-TR84 B08 to MAM), and the Einstein Foundation Berlin (EP-2017-393 to MAM). SG is a participant in the BIH-Charité Clinician Scientist Program funded by the Charité—Universitätsmedizin Berlin and the Berlin Institute of Health. SD is the recipient of an HRCMM (Heidelberg Research Center for Molecular Medicine) Career Development Fellowship.

## Conflict of Interest

MAM reports personal fees or grants from Boehringer Ingelheim, Arrowhead Pharmaceuticals, Vertex Pharmaceuticals, Santhera, Enterprise Therapeutics, and Antabio outside the submitted work.

The remaining authors declare that the research was conducted in the absence of any commercial or financial relationships that could be construed as a potential conflict of interest.

## Publisher’s Note

All claims expressed in this article are solely those of the authors and do not necessarily represent those of their affiliated organizations, or those of the publisher, the editors and the reviewers. Any product that may be evaluated in this article, or claim that may be made by its manufacturer, is not guaranteed or endorsed by the publisher.
