# Supplementary material for: Changes in Microbiome Dominance Are Associated With Declining Lung Function and Fluctuating Inflammation in People With Cystic Fibrosis
Source: Front Microbiol. 2022 May 13;13:885822. doi: 10.3389/fmicb.2022.885822 (PMC9136159; doi:10.3389/fmicb.2022.885822)
Supplement: Supplementary file 1 [file Data_Sheet_1.DOCX]

Supplementary Material

# Supplementary Methods

*Study cohort*

CF diagnosis was confirmed by established diagnostic criteria (Boeck et al., 2011; Farrell et al., 2017). From 26 patients with CF spontaneously expectorated sputum was collected during visits at the CF Center at the University Hospital Heidelberg. On the same day of sputum collection pulmonary function testing was performed according to ATS/ERS guidelines (Miller, 2015; Wagner, 2015) and normalization of FEV_1_ measures was done according to the global lung function initiative (Quanjer et al., 2012). Antibiotic (AB) status: 2 patients (7.7%) did not receive any AB treatment, 10 (38.5%) received inhaled ABs, 2 (7.7%) received inhaled and oral ABs and 12 patients received an alternating treatment consisting of inhaled and/or oral and/or IV Abs (Supplementary Figure S1). AB regimes of the study arms are given in Table 1. Of the 118 visits included, 59 were under inhaled ABs, 2 under oral ABs, 2 under IV ABs, 26 under inhaled and oral ABs, 4 under inhaled and IV ABs, 7 under inhaled/oral/IV ABs, 16 without ABs and 2 unknown. Patients were classified as exacerbated if at least 4 of the 12 criteria for antibiotic treatment are true: sputum changes (more, colour change, more viscous…); haemoptysis (new/more); increased cough; (Increased) dyspnea; malaise, exhaustion, lethargy; body temperature > 38°C; anorexia or weight loss; sinus pain or tension; altered sinunasal discharge; changes on clinical examination of the chest; worsening of pulmonary function by ≥ 10% from the previous findings; radiographic changes suggestive of pulmonary infection. A non-exacerbated visit was chosen as baseline visit.

*Sputum collection and sample pre-treatment for inflammatory biomarker analysis*

Mucus clumps were separated from saliva and incubated with 4 parts w/v of 10% Sputolysin (Merck, Darmstadt) for 15 min at room temperature on a rocking shaker. Afterwards, an equal volume of cold sterile PBS was added to stop the reaction. The solution was homogeneously mixed by carefully pipping up and down several times using a 10 mL Stripette (Corning, Corning, New York, USA). The mixture was filtered through a 100 µm and 40 µM cell strainer to obtain a solution free of mucus clumps and larger particles. The obtained mixture was centrifuged at 300 x *g* for 10 min at 4 °C. Afterwards, the supernatant fraction was aspirated and stored in aliquots at -80 °C until samples were further analysed. Aliquots stored for cytokine evaluation were supplemented with cOmplete™ Protease Inhibitor Cocktail (Roche, Basel, Switzerland) to prevent enzymatic degradation. The cell pellets were recovered in cold sterile PBS and counted using trypan blue staining.

*Inflammatory biomarker analysis*

As described previously (Frey et al., 2021a) inflammatory cytokines were assessed by Cytometric Bead Array (BD Bioscience, Franklin Lakes, New Jersey, USA), according to manufacturer’s protocols, IL-6 and TNF-α were measured pure using enhanced sensitivity assays, whereas IL‑1β and IL-8 samples were diluted 1:50 with sterile PBS. Anti-protease measurements were performed as duplicated using ELISA assays. Samples evaluated for NE/A1AT complex (Human PMN-Elastase ELISA Kit, Invitrogen, Carlsbad, California, USA) samples were diluted 1:100 with sterile PBS, for SLPI (Human Secretory Leucocyte Protease Inhibitor, Quantakine® ELISA (R&D Systems, Minneapolis, Minnesota, USA) were diluted 1:500 with sterile PBS and TIMP1 (Quantakine ELISA Human TIMP1 Immunoassay R&D Systems, Minneapolis, MN, USA) were diluted 1:100 with sterile PBS all ELISA procedures were performed according to manufactures protocol. ELISAs were read out using EnSpire 2300 (PerkinElmer, Waltham, Massachusetts, USA) plate reader. Free NE was measured as previously described (Frey et al., 2021b; Frey et al., 2021a), in brief, samples were diluted 1:500 and analysed in duplicates. 40 µL of the sample were placed in a black flat bottom 96 well ½ area plate (Corning Life Science, Kennebunk, Maine, USA) to which 10 µL master mix (containing 10 mM TRIS-HCl, 500 mM NaCl, pH 7.5 and 1 µM of NEmo-1 (Sirius Fine Chemicals, Bremen, Germany) were added the assay readout was started immediately. The FRET reporter was excited at 354 nm and fluorescence was recorded at 400 nm (for the donor) and 490 nm (for the receptor) every 60 sec for 50 cycles. Alongside the samples, a standard curve with known concentrations of Human Sputum Leucocyte Elastase (Elastin Products Company, Owensville, Missouri, USA) reaching from 0.5 µg/mL to 0.008 µg/mL in a 1:2 serial dilution was quantified. For each time point, the donor/acceptor ratio was calculated by dividing the readout at donor emission by the acceptor emission. The mean of the calculated ratios of the duplicates was plotted over time and the slope of the linear area was calculated. Acceptation criterium was to have at least 5 points within a linear range, samples and standards not fulfilling these criteria were excluded from further analysis. Slopes of known concentrations were plotted against the concentrations to generate a standard curve which was used to fit the slope of the samples. Finally, the fitted concentrations were multiplied by the dilutions of the samples to get the final concentration.

*Microbiome library preparation*

Spontaneously expectorated sputum specimens were directly taken to the local microbiology lab. Samples were stored at 4°C and processed within 24h. Samples were aliquoted (200 µl) and treated with PMA dye (Biotium Inc., Hayward, USA). PMA treatment modifies extracellular DNA from dead cells and avoids subsequent PCR amplification. 50 µM of PMA dye was added to the aliquot and incubated for 5 min in the dark. The samples were then exposed to light (650 Watt, 20 cm distance to the samples) on ice and shaking at 100 rpm for 5 min to cross-link PMA to DNA. Viable cells were pelleted by centrifugation at 5000 x g for 10 min. Supernatant was removed and cells were recovered in 200 µl of sterile PBS and stored at -20°C until DNA extraction. DNA extractions were performed using the QIAamp Mini Kit (QIAGEN, Hilden, Germany). Protease solution (7.2 mAU) and 200 µl of Buffer AL were added to 200 µl of the sample followed by a 15 sec vortex. Samples were incubated at 56°C for 10 min and then purified according to the manufacturer’s protocol. DNA was eluted by adding 100 µl of buffer AE to the column, incubation for 1 min at room temperature and centrifugation at 6000 x g for 1 min. Negative controls were performed by doing the extraction without clinical samples. The number of 16S copies was quantified by quantitative PCR (qPCR) using Unibac primer (forward: 5′-TGG AGC ATG TGG TTT AAT TCG A-3′; reverse: 5′-TGC GGG ACT TAA CCC AAC A-3′). PCR reactions were performed in 15 µl volumes composed of 1X Sybr-green mastermix (Life technology, Darmstadt, Germany), 50 pmol of each primer and 2 µl of DNA (or plasmid DNA standards). The thermal cycler conditions were: a first denaturation at 95°C for 20 sec, 40 amplification cycles (95°C for 3 sec, 60°C for 30 sec) and two final steps at 95°C for 15 sec and 60°C for 1 minute followed by melt curve analysis for specificity control. All reactions were performed in duplicates in a StepOnePlus Real-time PCR system (Applied Biosystems, Foster City, USA). Quantification of the 16S number of copies was performed by comparison to the cycle threshold value of a plasmid DNA standard which had been quantified by spectrophotometry.

DNA was amplified using universal bacterial primers flanking the V4 region (515F and 806R from Caporaso et al., 2012). Each primer was tagged with an individual barcode (each barcode had at least 3nt differences to the others) to assign the sequences to the samples. PCR reactions were performed in 25 µl volumes composed of Q5 High-Fidelity 1X Master Mix (New England Biolabs GmbH, Germany), 25 pmol of each primer and 2 µl of DNA. The thermal cycler (Primus 25, Peqlab Biotechnologie GmbH, Germany or FlexCycler², Analytik Jena AG, Germany) conditions were: a first denaturation at 94°C for 3 min, 30 amplification cycles (94°C for 45 sec, 50°C for 1 min and 72°C for 1 min 30 sec) and a final extension at 72°C for 10 minutes. Negative controls were performed using the negative control from the extraction step and using sterile water as template. For each run of sequencing (pool of 95 samples), an internal control was performed by amplifying a mock community sample containing genomic DNA from 20 bacterial strains in equimolar (even) ribosomal RNA operon counts (HMD-782D, BEI resources, ATCC, USA). PCR products were checked on Qiaxcel device for presence of amplicons. Amplicons were then ligated to Illumina by PCR and purified using Agencourt AMPure XP beads (Beckman Coulter, Krefeld, Germany) following the manufacturer’s instructions. Purified products were checked for concentration using Quant-iT™ PicoGreen™ dsDNA Assay Kit following manufacturer’s instructions. An equimolar mix of all PCR products was pooled and paired-end sequenced on an Illumina Miseq sequencing system with 600 cycles. Both negative controls were negative on the gel and library preparation resulted in no usable reads (percentage of reads in the run < 0.005% and 0 reads remained after quality control and chimera removal).

*Bioinformatics and Pre-Processing of the Data*

Raw reads were processed using the Dada2 package (v.1.12.1) (Callahan et al., 2016) to produce amplicon sequence variants (ASVs). The reads were filtered and trimmed using the following parameters: no ambiguities allowed; one error per read allowed; truncation of reads at first position with quality score less than two. Chimeras were removed following the default parameters. Taxonomic assignment of ASVs was performed using the ‘assignTaxonomy’ function in Dada2, which utilized the RDP Naïve Bayesian Classifier method (Wang et al., 2007) and training sets constructed from the Silva v128 database (Quast et al., 2013). Sequencing data and script are available in NCBI and figshare (PRJNA785129, https://figshare.com/s/de66d2124dc8687c1d97).

We constructed a phylogenetic tree of the ASVs using maximum likelihood analysis and a GTR+G+I nucleotide substitution model, in the phangorn package v2.5.5 (Schliep, 2011). The GTR+G+I nucleotide substitution model was chosen because it had the highest log likelihood and lowest Akaike Information Criterion (AIC) and Bayesian Information Criterion (BIC) among the models tested (Posada and Buckley, 2004). The tree was midpoint rooted using the phytools package (Revell, 2012).

The ASVs were filtered to remove those that were not assigned to the kingdom Bacteria, and those that were classified as class ‘Chloroplast’ or family ‘Mitochondria’. We also removed all ASVs that were only found in one sample, as these are likely to be erroneous (Goodrich et al., 2014). The extraction and PCR negative controls did not show successful amplification and the DNA quantification after library prep showed no DNA (<0.01ng), and thus no ASVs were removed based on being potential contaminants. After all filtering steps, 614 ASVs and 4,556,760 reads remained, with the number of reads per sample ranging from 7,457 to 106,576. Rarefaction curves, produced using the ‘rarecurve’ function in the package vegan (v. 2.5-6) (Oksanen J et al.), reached a plateau for every sample, which shows the bacterial communities were well sampled.

*Statistical Analysis*

All statistical analyses were performed in R v3.6.1 (R Core Team, 2017). The same analyses were performed on both datasets. For each sample, the bacterial communities were characterized by calculating species richness, α-diversity, β-diversity and dominance. Species richness was measured using Hill numbers when q=0, and α-diversity was measured using Hill numbers when q=1, which is equivalent to the exponential of Shannon’s index (M. O. Hill, 1973). The Hill numbers were calculated from the raw read counts using rarefaction/ extrapolation curves to account for differences in sampling depth among samples (Chao et al., 2014). For β-diversity, ASV abundances were converted to relative abundances to control for differences in sequencing depth and then weighted UniFrac distances were calculated between all samples, using the distance function in the package phyloseq (v1.28.0) (McMurdie and Holmes, 2013). The dominance of each community was determined as the relative abundance of the most abundant ASV in a sample.

A PERMANOVA with 9999 permutations was used to determine whether the lung bacterial community differed among patients, and was performed using the ‘adonis’ function in vegan (v.2.5-6) (Oksanen J et al.), using the weighted UniFrac distance matrix described above.

Linear mixed-effects models (LMMs) were used to assess the relationship between time and the response variables: species richness, α-diversity, biomass, lung function, and inflammation factors. For analysis of the 1 year data, α-diversity, biomass, IL-6, IL1-β, IL‑8, NE/A1AT complex, and TNF-α were log-transformed, while NE, SLPI and protein content were cube root transformed, and TIMP1 was square-root transformed, to ensure the residuals were normally distributed. The same transformations were used for the analysis of the 3+ year data, except SLPI was log-transformed and TIMP1 was cube root transformed. Time, gender, status (whether the sample was taken during an exacerbation or not) and whether the patient was receiving antibiotics were included as fixed effects in the models, while patient identity was included as a random effect. The LMMs were carried out in the package lme4 (v1.1-21) (Bates et al., 2015), and the Satterthwaite approximation was used to compute p-values, using the package lmerTest (v3.1-1) (Kuznetsova et al., 2017). Furthermore, the association of β-diversity and dominance with time was modelled using generalized linear mixed models (GLMMs) with a β‑distribution. The analysis was performed using the package glmmTMB (v1.0.2.1) (Brooks et al., 2017), with the same fixed and random effects as above.

In addition, the change in relative abundance over time of the genera and phyla with greater than 1% relative abundance, was investigated using either zero-inflated Gaussian mixed-effects models (ZIGMMs) or GLMMs with a β-distribution, depending on whether the data was zero-inflated. Separate models were implemented for each genus and phyla. For each ZIGMM model, the response variable was the arcsine square-root transformed relative abundances of the genus or phyla, while time, gender, status and whether antibiotics were being received were fixed effects, with patient identity as a random effect. The same fixed and random effects were used for the GLMMs. ZIGMMs were performed in the package NBZIMM (v1.0) (Xinyan Zhang and Nengjun Yi, 2020), while the GLMMs were performed using the package glmmTMB (v1.0.2.1) (Brooks et al., 2017). To account for multiple tests, the p-values were adjusted separately within each set of phyla and genera comparisons using the Benjamini-Hochberg method.

Furthermore, the relationship between the relative abundance of the top phyla, and the inflammation and clinical parameters was investigated using an LMM, with the clinical or inflammation factor as the response variable, and the phyla relative abundance, gender, status and whether antibiotics were received as the fixed effects. Patient ID was the random effect. The p-values were adjusted separately within the set of comparisons of each response variable using the Benjamini-Hochberg method, to account for multiple comparisons.

The relationship between the microbiota variables (α-diversity, β‑diversity, biomass and dominance), clinical variables (lung function and BMI) and inflammation variables within a patient over time was determined using four separate correlation analyses, which will be discussed below. Each of the correlation analyses examined the correlation of all of the variables measured in this study with each other, i.e. the correlations between α-diversity, β‑diversity, biomass, dominance, lung function, BMI, NE activity, and levels of TNF-α, IL-1β, IL-8, IL-6, protein content, SLPI and NE/A1AT complex.

In the first correlation analysis, we investigated whether the rate of change in a variable within a patient correlated with the rate of change in other variables. We created linear models for each patient, with time as an independent variable, and then one of the microbiota, clinical or inflammation parameters as the dependent variable. This produced a set of regression coefficients showing how the dependent variable varies with time for each patient. Correlation analysis was then used to assess the strength of the correlation between regression coefficients of different variables.

Secondly, we tested whether the stability of a variable within a patient was correlated across variables. Stability was defined as the mean pairwise distance between all samples within a patient. Therefore, for each variable, the mean pairwise distance was calculated for each patient, and the correlation between these stability values of different variables was determined.

Finally, we investigated whether α-diversity and lung function values at the patient baseline, correlated with the rate of change of the microbiota, clinical and inflammation parameters within a patient, as well as with the stability of β-diversity. The rate of change was calculated using regression coefficients, the same as for the first correlation analysis, and stability was calculated using the method from the second analysis.

The correlation coefficients for all three analyses were calculated using Spearman rank correlations, as this method does not assume linear relationships and is less sensitive to outliers (Hauke and Kossowski, 2011). Both the correlation coefficients and p-values were calculated using the ‘corr.test’ function in the package psych (v1.9.12.31) (Revelle W, 2019), which uses t-tests to determine the significance of each correlation. The Benjamini-Hochberg method was used to account for multiple comparisons, with p-values adjusted separately within each set of comparisons between α-diversity metrics, β-diversity, inflammation parameters and clinical parameters. The resulting correlations were visualized as networks using the packages ggraph (v2.04) (Pedersen, 2020) and igraph (v.1.7.4.2) (Csardi G and Nepusz T, 2006).

# Supplementary Results

*Overall microbiota composition of sputum samples from PwCF*

The microbial communities identified in the sputum of PwCF were dominated by the phyla Proteobacteria, Bacteroidetes, and Firmicutes, which collectively made up 89 % of all reads. *Pseudomonas* was by far the most abundant genus, followed by *Prevotella_7*, *Veillonella, Streptococcus, Haemophilus,* and *Staphylococcus*, with each of these genera making up over 5 % of all reads. At the sequence level, the top 10 ASVs comprised over half of all reads. The most prevalent genera were *Streptococcus, Rothia, Veillonella, Prevotella_7, Prevotella,* and *Capnocytophaga,* whilst *Pseudomonas* was only present in 59 % of samples. In line with previous studies, there were significant differences among patients in their bacterial communities, with 67 % of the variation in the microbiota explained by the patient (PERMANOVA: R^2^=0.666, p<0.001; Supplementary Figure S2) (Stressmann et al., 2012; Zhao et al., 2012).

# Supplementary tables

**Supplementary Table S1:** CFTR genotypes and pancreatic status based on elastase levels in stool of patients with cystic fibrosis

| **Pancreatic insufficient** | | **Pancreatic sufficient** | |
| --- | --- | --- | --- |
| ***CFTR* genotype** | **subjects (n)** | ***CFTR* genotype** | **subjects (n)** |
| F508del / F508del | 11 | 1717-1G>A / R764X | 1 |
| F508del / 1811+17T>G | 1 | G542X / X | 1 |
| F508del / 3905insT | 1 | I336K / c.3964-7A>G | 1 |
| F508del / 711+ 3A>G | 1 |  |  |
| F508del / 3600+2insT | 1 |  |  |
| F508del / CFTRdel17 (2.5Kb) | 1 |  |  |
| F508del / E403D | 1 |  |  |
| F508del / G551D | 1 |  |  |
| F508del / I507del | 1 |  |  |
| F508del / Q220X | 1 |  |  |
| F508del / R347P | 1 |  |  |
| F508del / T1299I | 1 |  |  |
| F508del / W1282X | 1 |  |  |
| 174delA / R347P | 1 |  |  |

**Supplementary Table S2:** The relationship of the microbiome, clinical and taxonomic variables with exacerbation events, total antibiotic use, inhaled antibiotic use, oral antibiotic use and IV antibiotic use, in both the 1 and 3+ years study. The p-values were calculated using either LMMs, GLMMs or ZIGMMs as outlined above. The phyla and genera p-values were adjusted for multiple comparisons using the Benjamini-Hochberg method.

|  | **Exacerbation** | | **Total Antibiotics** | | **Inhaled** | | **Oral** | | **IV** | |
| --- | --- | --- | --- | --- | --- | --- | --- | --- | --- | --- |
|  | Effect Size  (Standard Error) | P-  value | Effect Size  (Standard Error) | P-  value | Effect Size  (Standard Error) | P-  value | Effect Size  (Standard Error) | P-  value | Effect Size  (Standard Error) | P-value |
|  |  |  |  | **1 year** | |  |  |  |  |  |
| **Diversity** |  |  |  |  |  |  |  |  |  |  |
| Richness | -11.46 (17.50) | 0.517 | -2.48 (10.14) | 0.808 | -4.75 (9.33) | 0.613 | -4.29 (8.87) | 0.631 | -2.68 (11.32) | 0.814 |
| α-diversity | -0.213 (0.495) | 0.671 | 0.145 (0.275) | 0.601 | -0.074 (0.257) | 0.774 | 0.127 (0.246) | 0.608 | -0.0008 (0.319) | 0.998 |
| β-diversity | -0.194 (0.312) | 0.534 | 0.149 (0.252) | 0.555 | 0.094 (0.214) | 0.661 | -0.248 (0.200) | 0.216 | 0.038 (0.223) | 0.865 |
| Dominance | 0.414 (0.618) | 0.503 | 0.127 (0.420) | 0.763 | 0.365 (0.333) | 0.273 | -0.237 (0.319) | 0.458 | -0.066 (0.402) | 0.870 |
| Biomass | -1.99 (1.46) | 0.180 | 0.628 (0.695) | 0.373 | 0.096 (0.672) | 0.887 | 0.043 (0.66) | 0.948 | 1.49 (0.927) | 0.117 |
| **Clinical** |  |  |  |  |  |  |  |  |  |  |
| FEV_1_% predicted | -4.59 (6.26) | 0.469 | **-9.40 (3.58)** | **0.013** | **-8.06 (3.26)** | **0.019** | 1.06 (3.13) | 0.738 | -3.85 (4.44) | 0.393 |
| **Inflammation** |  |  |  |  |  |  |  |  |  |  |
| Protein Content | **-3.49 (1.71)** | **0.048** | 0.828 (0.809) | 0.316 | -0.195 (0.742) | 0.794 | -1.17 (0.741) | 0.124 | **3.14 (1.08)** | **0.006** |
| IL-1β | -0.250 (1.50) | 0.868 | 1.40 (0.736) | 0.064 | 0.533 (0.733) | 0.471 | -0.153 (0.707) | 0.830 | 0.605 (0.957) | 0.531 |
| IL-6 | 1.43 (0.915) | 0.124 | 0.493 (0.431) | 0.262 | 0.441 (0.399) | 0.279 | -0.608 (0.398) | 0.135 | -0.309 (0.576) | 0.594 |
| IL-8 | -0.260 (1.23) | 0.834 | **1.40 (0.585)** | **0.022** | 0.939 (0.593) | 0.121 | -0.764 (0.574) | 0.190 | 0.708 (0.788) | 0.374 |
| NE/A1AT Complex | -1.13 (0.839) | 0.184 | 0.654 (0.404) | 0.119 | 0.399 (0.385) | 0.306 | -0.657 (0.380) | 0.092 | **1.64 (0.532)** | **0.004** |
| NE | **-3.98 (1.35)** | **0.005** | 0.960 (0.568) | 0.101 | 0.903 (0.495) | 0.075 | -0.879 (0.504) | 0.089 | **2.49 (0.661)** | **<0.001** |
| SLPI | 0.087 (1.76) | 0.961 | 0.826 (0.847) | 0.336 | 1.23 (0.802) | 0.134 | -0.391 (0.788) | 0.622 | -0.648 (1.12) | 0.564 |
| TIMP1 | -3.73 (2.79) | 0.190 | 2.69 (1.48) | 0.076 | 1.60 (1.40) | 0.259 | -2.39 (1.34) | 0.082 | 1.89 (1.79) | 0.297 |
| TNF-α | -0.125 (1.13) | 0.913 | 1.09 (0.540) | 0.051 | 0.222 (0.550) | 0.688 | -0.393 (0.532) | 0.463 | 0.238 (0.724) | 0.744 |
| **Phyla** |  |  |  |  |  |  |  |  |  |  |
| Proteobacteria | 1.22 (0.708) | 0.258 | 0.408 (0.401) | 0.698 | 0.368 (0.362) | 0.807 | -0.457 (0.362) | 0.618 | -0.314 (0.456) | 0.491 |
| Bacteroidetes | -1.18 (0.704) | 0.258 | -0.098 (0.352) | 0.955 | 0.080 (0.302) | 0.807 | 0.161 (0.304) | 0.896 | 0.438 (0.367) | 0.233 |
| Firmicutes | 0.164 (0.633) | 0.867 | -0.385 (0.291) | 0.698 | -0.687 (0.272) | 0.072 | 0.665 (0.267) | 0.072 | -0.319 (0.399) | 0.425 |
| Actinobacteria | -0.154 (0.703) | 0.867 | -0.036 (0.353) | 0.955 | 0.124 (0.332) | 0.807 | -0.031 (0.305) | 0.918 | 0.093 (0.489) | 0.849 |
| Fusobacteria | -0.012 (0.073) | 0.867 | 0.002 (0.039) | 0.955 | -0.009 (0.037) | 0.807 | -0.011 (0.035) | 0.917 | 0.008 (0.047) | 0.863 |
| Tenericutes | -0.025 (0.016) | 0.258 | -0.007 (0.008) | 0.698 | -0.003 (0.008) | 0.807 | 0.004 (0.007) | 0.896 | 0.005 (0.010) | 0.645 |
| **Genera** |  |  |  |  |  |  |  |  |  |  |
| *Pseudomonas* | 0.140 (0.160) | 0.732 | 0.054 (0.103) | 0.87 | 0.198 (0.087) | 0.39 | -0.110 (0.082) | 0.735 | -0.042 (0.103) | 0.931 |
| *Prevotella_7* | -0.031 (0.117) | 0.941 | 0.030 (0.066) | 0.87 | 0.049 (0.061) | 0.624 | 0.0007 (0.058) | 0.992 | 0.024 (0.076) | 0.931 |
| *Veillonella* | 0.001 (0.645) | 0.988 | 0.069 (0.329) | 0.984 | -0.014 (0.331) | 0.967 | 0.392 (0.273) | 0.735 | -0.062 (0.352) | 0.931 |
| *Streptococcus* | -0.589 (0.634) | 0.732 | -0.028 (0.245) | 0.985 | -0.096 (0.253) | 0.762 | 0.225 (0.237) | 0.735 | 0.294 (0.350) | 0.931 |
| *Haemophilus* | -0.101 (0.150) | 0.732 | 0.101 (0.082) | 0.735 | -0.078 (0.077) | 0.598 | 0.059 (0.074) | 0.735 | 0.047 (0.096) | 0.931 |
| *Staphylococcus* | 0.084 (0.120) | 0.732 | -0.066 (0.070) | 0.767 | -0.086 (0.063) | 0.543 | 0.082 (0.060) | 0.735 | -0.084 (0.077) | 0.931 |
| *Prevotella* | -0.113 (0.086) | 0.732 | 0.047 (0.049) | 0.767 | 0.056 (0.044) | 0.543 | 0.019 (0.042) | 0.939 | 0.116 (0.055) | 0.585 |
| *Neisseria* | 0.327 (0.124) | 0.169 | -0.094 (0.055) | 0.416 | -0.088 (0.055) | 0.543 | -0.050 (0.055) | 0.735 | -0.102 (0.078) | 0.931 |
| *Rothia* | -0.113 (0.706) | 0.946 | -0.002 (0.363) | 0.996 | 0.172 (0.340) | 0.747 | -0.024 (0.310) | 0.992 | 0.141 (0.504) | 0.931 |
| *Porphyromonas* | -0.060 (0.086) | 0.732 | -0.099 (0.044) | 0.416 | -0.037 (0.044) | 0.624 | 0.030 (0.042) | 0.735 | -0.005 (0.055) | 0.931 |
| *Alloprevotella* | -0.070 (0.071) | 0.732 | -0.016 (0.037) | 0.87 | -0.048 (0.035) | 0.543 | 0.033 (0.034) | 0.735 | 0.004 (0.045) | 0.931 |
| *Gemella* | -0.122 (0.082) | 0.732 | 0.030 (0.041) | 0.87 | 0.019 (0.040) | 0.747 | -0.009 (0.039) | 0.992 | -0.023 (0.052) | 0.931 |
| *Stenotrophomonas* | 0.059 (0.112) | 0.783 | -0.112 (0.061) | 0.416 | -0.061 (0.058) | 0.598 | -0.0005 (0.056) | 0.992 | -0.018 (0.072) | 0.931 |
|  |  |  |  | **3+ years** | |  |  |  |  |  |
| **Diversity** |  |  |  |  |  |  |  |  |  |  |
| Richness | 11.34 (12.44) | 0.365 | -1.71 (10.97) | 0.876 | 3.15 (8.07) | 0.698 | **-14.43 (6.02)** | **0.019** | -7.02 (8.30) | 0.400 |
| α-diversity | 0.366 (0.364) | 0.319 | 0.399 (0.318) | 0.214 | 0.289 (0.237) | 0.226 | -0.176 (0.177) | 0.324 | -0.402 (0.243) | 0.102 |
| β-diversity | -0.057 (0.327) | 0.861 | 0.141 (0.332) | 0.672 | 0.057 (0.237) | 0.810 | -0.117 (0.173) | 0.498 | 0.164 (0.224) | 0.463 |
| Dominance | -0.571 (0.410) | 0.164 | -0.306 (0.387) | 0.429 | -0.180 (0.280) | 0.521 | 0.114 (0.203) | 0.574 | **0.628 (0.270)** | **0.020** |
| Biomass | 0.0008 (1.11) | 0.999 | 0.487 (0.882) | 0.583 | -0.484 (0.694) | 0.487 | -0.504 (0.510) | 0.326 | 0.786 (0.732) | 0.287 |
| **Clinical** |  |  |  |  |  |  |  |  |  |  |
| FEV_1_% predicted | -1.70 (4.50) | 0.707 | -5.16 (4.07) | 0.209 | -0.028 (2.97) | 0.993 | -0.638 (2.33) | 0.785 | **-6.46 (3.18)** | **0.047** |
| **Inflammation** |  |  |  |  |  |  |  |  |  |  |
| Protein Content | **-2.39 (1.12)** | **0.036** | 0.777 (0.877) | 0.379 | -0.906 (0.661) | 0.175 | 0.565 (0.495) | 0.258 | **1.59 (0.731)** | **0.033** |
| IL-1β | -0.840 (0.863) | 0.334 | 0.502 (0.676) | 0.460 | -0.410 (0.521) | 0.434 | 0.504 (0.392) | 0.204 | 0.545 (0.567) | 0.340 |
| IL-6 | 0.499 (0.707) | 0.482 | 0.391 (0.603) | 0.518 | -0.058 (0.458) | 0.900 | -0.488 (0.351) | 0.169 | 0.108 (0.471) | 0.819 |
| IL-8 | -0.460 (0.643) | 0.477 | 0.684 (0.497) | 0.173 | 0.076 (0.391) | 0.846 | 0.176 (0.295) | 0.552 | 0.411 (0.423) | 0.335 |
| NE/A1AT Complex | -0.814 (0.513) | 0.118 | 0.013 (0.487) | 0.979 | -0.555 (0.336) | 0.102 | -0.144 (0.246) | 0.560 | **1.05 (0.343)** | **0.003** |
| NE | -1.60 (0.920) | 0.087 | 0.462 (0.624) | 0.461 | -0.088 (0.494) | 0.858 | 0.391 (0.363) | 0.286 | 0.957 (0.537) | 0.079 |
| SLPI | -0.302 (0.896) | 0.737 | -0.402 (0.638) | 0.531 | 0.149 (0.519) | 0.775 | -0.052 (0.385) | 0.892 | -0.318 (0.583) | 0.587 |
| TIMP1 | -0.817 (0.658) | 0.219 | -0.026 (0.462) | 0.956 | -0.002 (0.374) | 0.997 | -0.205 (0.280) | 0.467 | 0.142 (0.427) | 0.741 |
| TNF-α | -0.415 (0.765) | 0.589 | 0.571 (0.616) | 0.357 | -0.399 (0.476) | 0.404 | 0.374 (0.361) | 0.303 | 0.306 (0.506) | 0.547 |
| **Phyla** |  |  |  |  |  |  |  |  |  |  |
| Proteobacteria | 0.395 (0.632) | 0.929 | 0.320 (0.549) | 0.672 | -0.055 (0.407) | 0.892 | -0.424 (0.323) | 0.284 | 0.361 (0.398) | 0.588 |
| Bacteroidetes | -0.167 (0.491) | 0.929 | 0.023 (0.377) | 0.952 | 0.366 (0.298) | 0.330 | -0.313 (0.226) | 0.284 | -0.080 (0.302) | 0.792 |
| Firmicutes | 0.040 (0.451) | 0.929 | -0.470 (0.324) | 0.412 | -0.419 (0.255) | 0.202 | 0.444 (0.199) | 0.156 | -0.379 (0.322) | 0.588 |
| Actinobacteria | -0.413 (0.434) | 0.929 | 0.437 (0.345) | 0.412 | 0.485 (0.281) | 0.202 | 0.008 (0.185) | 0.964 | 0.260 (0.306) | 0.588 |
| Fusobacteria | 0.024 (0.064) | 0.929 | -0.050 (0.052) | 0.506 | -0.027 (0.040) | 0.593 | -0.040 (0.030) | 0.284 | -0.026 (0.042) | 0.655 |
| Tenericutes | 0.008 (0.033) | 0.929 | 0.068 (0.031) | 0.18 | 0.043 (0.023) | 0.202 | 0.020 (0.017) | 0.289 | -0.022 (0.022) | 0.588 |
| **Genera** |  |  |  |  |  |  |  |  |  |  |
| *Pseudomonas* | -0.054 (0.131 | 0.902 | -0.046 (0.120) | 0.810 | 0.058 (0.088) | 0.600 | -0.068 (0.066) | 0.652 | 0.186 (0.088) | 0.494 |
| *Prevotella_7* | 0.024 (0.084) | 0.902 | 0.040 (0.066) | 0.810 | 0.041 (0.052) | 0.562 | -0.019 (0.038) | 0.849 | 0.009 (0.055) | 0.872 |
| *Veillonella* | 0.326 (0.435) | 0.902 | -0.077 (0.321) | 0.810 | -0.150 (0.266) | 0.620 | 0.302 (0.225) | 0.471 | 0.051 (0.300) | 0.872 |
| *Streptococcus* | -0.305 (0.529) | 0.902 | 0.316 (0.326) | 0.724 | 0.260 (0.272) | 0.551 | 0.293 (0.190) | 0.403 | -0.117 (0.330) | 0.854 |
| *Haemophilus* | 0.017 (0.139) | 0.902 | 0.052 (0.112) | 0.81 | -0.136 (0.086) | 0.403 | -0.042 (0.064) | 0.849 | -0.067 (0.092) | 0.761 |
| *Staphylococcus* | 0.025 (0.084) | 0.902 | **-0.233 (0.074)** | **0.026** | -0.167 (0.055) | 0.052 | 0.070 (0.041) | 0.403 | -0.034 (0.056) | 0.781 |
| *Prevotella* | -0.030 (0.063) | 0.902 | 0.032 (0.055) | 0.81 | 0.060 (0.041) | 0.403 | -0.047 (0.030) | 0.403 | 0.044 (0.042) | 0.659 |
| *Neisseria* | **0.312 (0.088)** | **0.009** | -0.022 (0.063) | 0.81 | -0.044 (0.052) | 0.562 | -0.011 (0.038) | 0.849 | -0.093 (0.057) | 0.653 |
| *Rothia* | -0.430 (0.444) | 0.902 | 0.431 (0.349) | 0.564 | 0.478 (0.284) | 0.403 | 0.030 (0.189) | 0.874 | 0.252 (0.312) | 0.761 |
| *Porphyromonas* | 0.047 (0.067) | 0.902 | -0.134 (0.053) | 0.091 | -0.047 (0.042) | 0.551 | -0.009 (0.031) | 0.849 | -0.057 (0.044) | 0.653 |
| *Alloprevotella* | 0.014 (0.058) | 0.902 | 0.105 (0.048) | 0.143 | 0.052 (0.036) | 0.403 | -0.064 (0.027) | 0.273 | -0.018 (0.038) | 0.835 |
| *Gemella* | -0.057 (0.057) | 0.902 | 0.054 (0.042) | 0.564 | 0.032 (0.033) | 0.551 | 0.008 (0.024) | 0.849 | -0.041 (0.037) | 0.659 |
| *Stenotrophomonas* | -0.006 (0.035) | 0.902 | -0.010 (0.033) | 0.81 | 0.007 (0.024) | 0.761 | -0.006 (0.018) | 0.849 | 0.033 (0.024) | 0.653 |

**Supplementary figures**

**
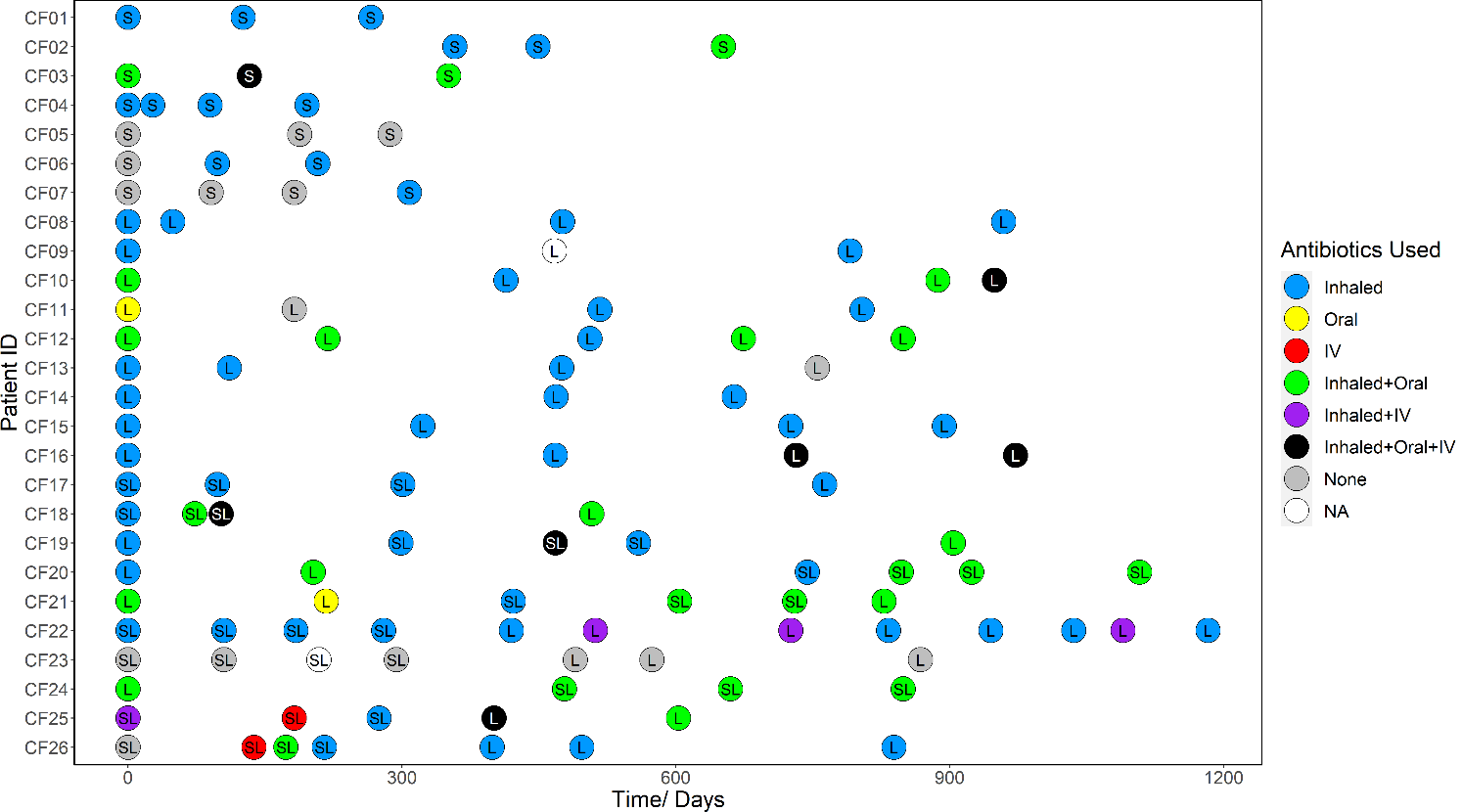
**

**Supplementary Figure S1.** The antibiotics used by a patient at each sampling time point. The colour of the circles relates to the type of antibiotic used, with grey meaning no antibiotic was taken, and white meaning there was no information on antibiotic use.

S= 1-year samples; L= 3+ years samples; SL= used in both the 1-year and 3+ years study.


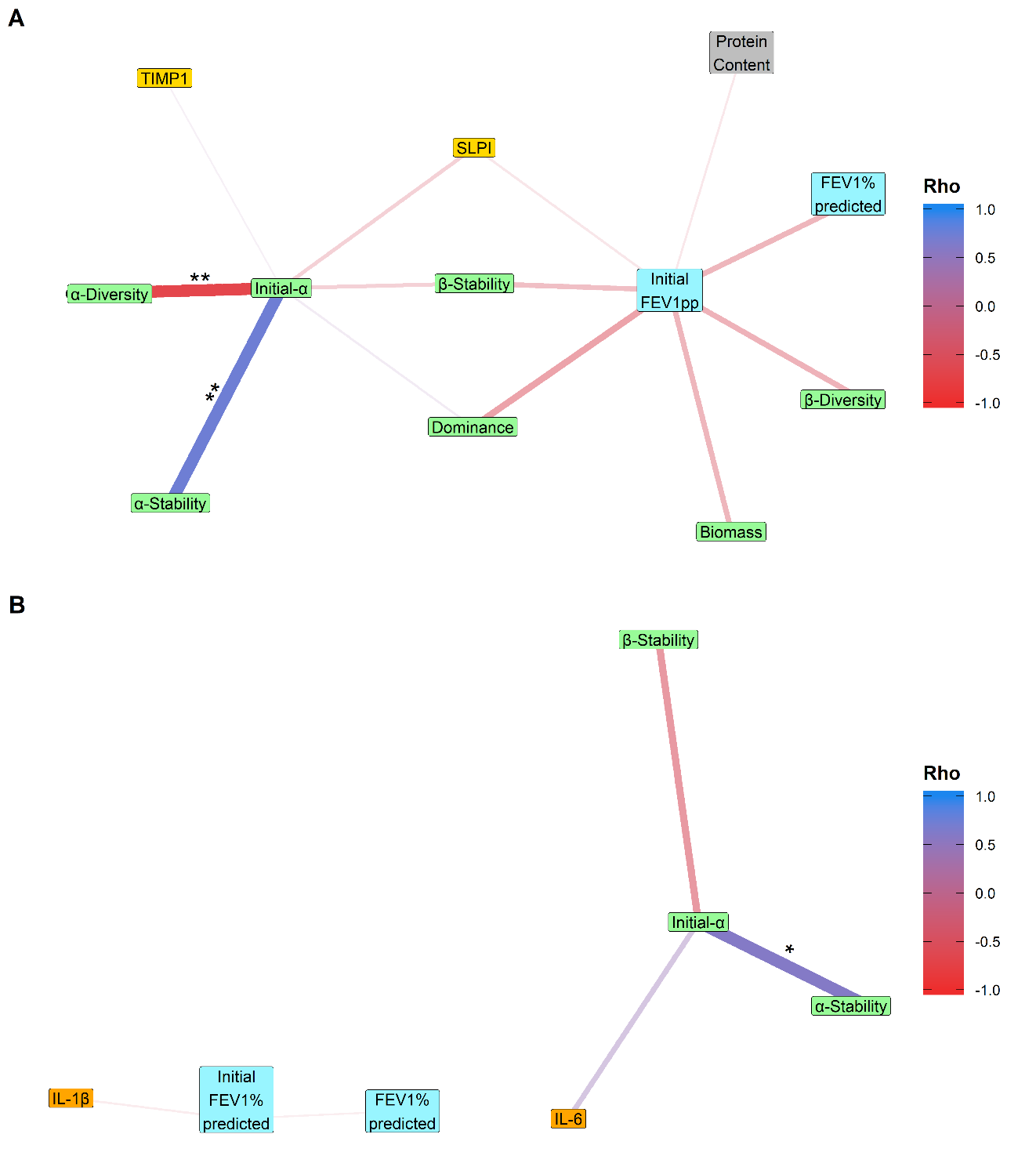


**Supplementary Figure S2.**  Correlations between the value of α-diversity and FEV_1_ % predicted at baseline of a patient, with the temporal changes of the microbiota (green), clinical (blue), pro-inflammatory parameters (orange), anti-protease (yellow) and general (grey) variables, as well as with the stability of α- and β-diversity. A) Represents the 1-year dataset, while B) represents the 3+ year’s dataset. The size of the Spearman rank correlation coefficient (Rho) is represented by the width and color of the edges, and only correlations with a Rho greater than 0.3 or smaller than -0.3 are shown. P-values were adjusted using the Benjamini-Hochberg method. P-values are indicated as: * <0.05; ** ≤0.01 and *** ≤0.001.


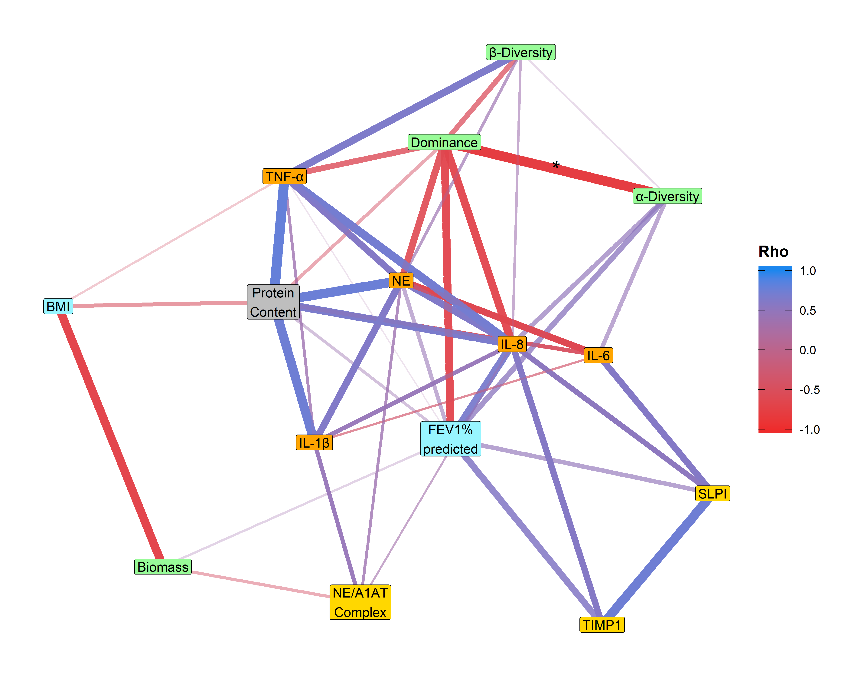


**Supplementary Figure 3.** Correlations between the rate of change ofvariables in the 3+ year cohort excluding patients dominated by *Pseudomonas aeruginosa*. For each variable, the relationship between that variable and time was determined within each patient, and the resulting regression coefficients were correlated across variables. Microbiota are given in green, clinical in blue, pro-inflammatory in orange, anti-protease in yellow and general parameters in grey. The size of the Spearman rank correlation coefficient (Rho) is represented by the width and color of the edges, and only correlations with a Rho greater than 0.3 or smaller than -0.3 are shown. P values were adjusted using the Benjamini-Hochberg method. P-values indicated as: * <0.05; ** ≤0.01 and *** ≤0.001.


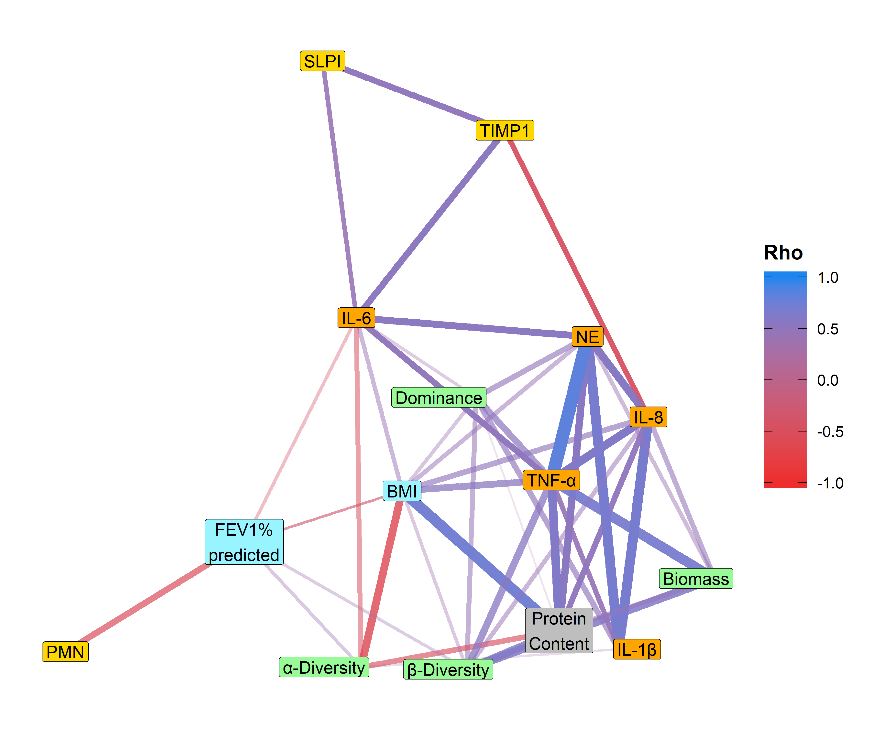
**Supplementary Figure 4.** Correlations between the intra-patient stability of variables in the 3+ year cohort excluding patients dominated by *Pseudomonas aeruginosa*. For each variable, intra-patient stability was calculated as the average pairwise distance between samples within a patient. These average distances were then correlated across variables. Microbiota are given in green, clinical in blue, pro-inflammatory in orange, anti-protease in yellow and general parameters in grey. The size of the Spearman rank correlation coefficient (Rho) is represented by the width and color of the edges, and only correlations with a Rho greater than 0.3 or smaller than -0.3 are shown. P values were adjusted using the Benjamini-Hochberg method. P-values indicated as: * <0.05; ** ≤0.01 and *** ≤0.001.

**Supplementary references**

Bates, D., Mächler, M., Bolker, B., and Walker, S. (2015). Fitting Linear Mixed-Effects Models Using lme4. *J Stat Soft* 67. doi: 10.18637/jss.v067.i01

Boeck, K. de, Derichs, N., Fajac, I., Jonge, H. R. de, Bronsveld, I., Sermet, I., et al. (2011). New clinical diagnostic procedures for cystic fibrosis in Europe. *J Cyst Fibros* 10, S53-S66. doi: 10.1016/s1569-1993(11)60009-x

Brooks, M. E., Kristensen, K., van Benthem, K. J., Magnusson, A., Berg, C. W., Nielsen, A., et al. (2017). glmmTMB balances speed and flexibility among packages for zero-inflated generalized linear mixed modeling. *R J* 9, 378-400. doi: 10.3929/ethz-b-000240890

Callahan, B. J., McMurdieE, P. J., Rosen, M. J., Han, A. W., Johnson, A. J. A., and Holmes, S. P. (2016). DADA2: High-resolution sample inference from Illumina amplicon data. *Nat Methods* 13, 581–583. doi: 10.1038/nmeth.3869

Caporaso, J. G., Lauber, C. L., Walters, W. A., Berg-Lyons, D., Huntley, J., Fierer, N., et al. (2012). Ultra-high-throughput microbial community analysis on the Illumina HiSeq and MiSeq platforms. *ISME J* 6, 1621–1624. doi: 10.1038/ismej.2012.8

Chao, A., Gotelli, N. J., Hsieh, T. C., Sander, E. L., Ma, K. H., Colwell, R. K., et al. (2014). Rarefaction and extrapolation with Hill numbers: a framework for sampling and estimation in species diversity studies. *Ecol Monogr* 84, 45–67. doi: 10.1890/13-0133.1

Csardi G, and Nepusz T (2006). The igraph software package for complex network research. *Int J Complex Syst* 1695.

Farrell, P. M., White, T. B., Ren, C. L., Hempstead, S. E., Accurso, F., Derichs, N., et al. (2017). Diagnosis of Cystic Fibrosis: Consensus Guidelines from the Cystic Fibrosis Foundation. *J Pediatr* 181S, S4-S15.e1. doi: 10.1016/j.jpeds.2016.09.064

Frey, D. L., Boutin, S., Dittrich, S. A., Graeber, S. Y., Stahl, M., Wege, S., et al. (2021a). Relationship between airway dysbiosis, inflammation and lung function in adults with cystic fibrosis. *J Cyst Fibros*. doi: 10.1016/j.jcf.2020.12.022

Frey, D. L., Guerra, M., Mall, M. A., and Schultz, C. (2021b). Monitoring Neutrophil Elastase and Cathepsin G Activity in Human Sputum Samples. *J Vis Exp*, e62193. doi: 10.3791/62193

Goodrich, J. K., Di Rienzi, S. C., Poole, A. C., Koren, O., Walters, W. A., Caporaso, J. G., et al. (2014). Conducting a microbiome study. *Cell* 158, 250–262. doi: 10.1016/j.cell.2014.06.037

Hauke, J., and Kossowski, T. (2011). Comparison of Values of Pearson's and Spearman's Correlation Coefficients on the Same Sets of Data. *Quaest Geogr* 30, 87–93. doi: 10.2478/v10117-011-0021-1

Kuznetsova, A., Brockhoff, P. B., and Christensen, R. H. B. (2017). lmerTest Package: Tests in Linear Mixed Effects Models. *J Stat Soft* 82. doi: 10.18637/jss.v082.i13

M. O. Hill (1973). Diversity and Evenness: A Unifying Notation and Its Consequences. *Ecology* 54, 427–432. doi: 10.2307/1934352

McMurdie, P. J., and Holmes, S. (2013). phyloseq: an R package for reproducible interactive analysis and graphics of microbiome census data. *PLOS One* 8, e61217. doi: 10.1371/journal.pone.0061217

Miller, M. R. (2015). Defining airflow obstruction. *Eur Respir J* 45, 560. doi: 10.1183/09031936.00157114

Oksanen J, Blanchet FG, Friendly M, Kindt R, Legendre P, Mcglinn D, et al. vegan: Community Ecology Package 2018.

Pedersen, T. L. (2020). ggraph: An implementation of grammar of graphics for graphs and networks.

Posada, D., and Buckley, T. R. (2004). Model selection and model averaging in phylogenetics: advantages of akaike information criterion and bayesian approaches over likelihood ratio tests. *Syst Biol* 53, 793–808. doi: 10.1080/10635150490522304

Quanjer, P. H., Stanojevic, S., Cole, T. J., Baur, X., Hall, G. L., Culver, B. H., et al. (2012). Multi-ethnic reference values for spirometry for the 3-95-yr age range: The global lung function 2012 equations. *Eur Respir J* 40, 1324–1343. doi: 10.1183/09031936.00080312

Quast, C., Pruesse, E., Yilmaz, P., Gerken, J., Schweer, T., Yarza, P., et al. (2013). The SILVA ribosomal RNA gene database project: improved data processing and web-based tools. *Nucleic Acids Res* 41, D590-6. doi: 10.1093/nar/gks1219

R Core Team (2017). A language and environment for statistical computing: Vienna, Austria: R Foundation for Statistical Computing.

Revell, L. J. (2012). phytools: An R package for phylogenetic comparative biology (and other things). *Methods Ecol Evol* 3, 217–223. doi: 10.1111/j.2041-210X.2011.00169.x

Revelle W (2019). psych: Procedures for Psychological, Psychometric, and Personality Research: R package version 1.9.12.31.

Schliep, K. P. (2011). phangorn: phylogenetic analysis in R. *Bioinformatics* 27, 592–593. doi: 10.1093/bioinformatics/btq706

Stressmann, F. A., Rogers, G. B., van der Gast, C. J., Marsh, P., Vermeer, L. S., Carroll, M. P., et al. (2012). Long-term cultivation-independent microbial diversity analysis demonstrates that bacterial communities infecting the adult cystic fibrosis lung show stability and resilience. *Thorax* 67, 867–873. doi: 10.1136/thoraxjnl-2011-200932

Wagner, P. D. (2015). The physiological basis of pulmonary gas exchange: implications for clinical interpretation of arterial blood gases. *Eur Respir J* 45, 227–243. doi: 10.1183/09031936.00039214

Wang, Q., Garrity, G. M., Tiedje, J. M., and Cole, J. R. (2007). Naive Bayesian classifier for rapid assignment of rRNA sequences into the new bacterial taxonomy. *Appl Environ Microbiol* 73, 5261–5267. doi: 10.1128/AEM.00062-07

Xinyan Zhang, and Nengjun Yi (2020). NBZIMM: negative binomial and zero-inflated mixed models, with application to microbiome/metagenomics data analysis. *BMC Bioinform.* 21, 1–19. doi: 10.1186/s12859-020-03803-z

Zhao, J., Schloss, P. D., Kalikin, L. M., Carmody, L. A., Foster, B. K., Petrosino, J. F., et al. (2012). Decade-long bacterial community dynamics in cystic fibrosis airways. *Proc Natl Acad Sci* 109, 5809–5814. doi: 10.1073/pnas.1120577109
